# Clinical impact of screening computed tomography in extracorporeal membrane oxygenation: a retrospective cohort study

**DOI:** 10.1186/s13613-023-01187-w

**Published:** 2023-09-26

**Authors:** Patrick D. Collins, Lorenzo Giosa, Sushil Kathar, Valentina Camarda, Filippo Palmesino, Darshan Eshwar, Nicholas A. Barrett, Andrew Retter, Francesco Vasques, Barnaby Sanderson, Sze M. Mak, Louise Rose, Luigi Camporota

**Affiliations:** 1 Department of Critical Care Medicine, Guy’s and St Thomas’ NHS Foundation Trust, St Thomas’ Hospital, Westminster Bridge Road, London, SE1 7EH UK; 2 Centre for Human and Applied Physiological Sciences, School of Basic and Medical Biosciences, King’s College London, London, UK; 3 Department of Radiology, Guy’s and St Thomas’ NHS Foundation Trust, London, UK; 4 Florence Nightingale Faculty of Nursing, Midwifery and Palliative Care, King’s College London, London, UK

**Keywords:** Veno-venous extracorporeal membrane oxygenation, Computed tomography, Respiratory distress syndrome, Acute

## Abstract

**Background:**

Data on the prevalence and clinical impact of extrapulmonary findings at screening computed tomography (CT) on initiation of veno-venous extracorporeal membrane oxygenation (V-V ECMO) are limited. We aimed to identify the prevalence of extrapulmonary findings on screening CT following V-V ECMO initiation. We hypothesized that extrapulmonary findings would influence clinical management and outcome.

**Methods:**

Retrospective analysis (2011–2021) of admission screening CT including head, abdomen and pelvis with contrast of consecutive patients on initiation of V-V ECMO. CT findings identified by the attending consultant radiologist were extracted. Demographics, admission physiological and laboratory data, clinical decision-making following CT and ECMO ICU mortality were recorded from the electronic medical record. We used multivariable logistic regression and Kaplan–Meier curves to evaluate associations between extrapulmonary findings and ECMO ICU mortality.

**Results:**

Of the 833 patients receiving V-V ECMO, 761 underwent routine admission CT (91.4%). ECMO ICU length of stay was 19 days (IQR 12–23); ICU mortality at the ECMO centre was 18.9%. An incidental extrapulmonary finding was reported in 227 patients (29.8%), leading to an invasive procedure in 12/227 cases (5.3%) and a change in medical management (mainly in anticoagulation strategy) in 119/227 (52.4%). Extrapulmonary findings associated with mortality were intracranial haemorrhage (OR 2.34 (95% CI 1.31–4.12), cerebral infarction (OR 3.59 (95% CI 1.26–9.86) and colitis (OR 2.80 (95% CI 1.35–5.67).

**Conclusions:**

Screening CT frequently identifies extrapulmonary findings of clinical significance. Newly detected intracranial haemorrhage, cerebral infarction and colitis were associated with increased ICU mortality.

**Supplementary Information:**

The online version contains supplementary material available at 10.1186/s13613-023-01187-w.

## Introduction

Veno-Venous Extracorporeal Membrane Oxygenation (V-V ECMO) is used to support patients with severe respiratory failure, such as the acute respiratory distress syndrome (ARDS) [1–4]. These patients may have extrapulmonary causes or consequences of their critical illness. However, there is limited evidence regarding the use of routine screening of extrapulmonary pathology on V-V ECMO cannulation. Consequently, there is variation in practice and outcomes among centres, which may reflect different screening procedures and processes of care [5].

At our centre, one of six specialized severe respiratory failure centres commissioned by the National Health Service in the UK to provide V-V ECMO [6], the routine care of patients undergoing ECMO has included a comprehensive screening computed tomography (CT) of the head, chest, abdomen and pelvis upon return to the hospital following mobile ECMO retrieval (1–6 h post ECMO cannulation) [7]. Thoracic CT has recognized diagnostic and prognostic yield in the management of patients with severe respiratory failure [8, 9]. In contrast, the systematic use of an extrapulmonary screening CT in V-V ECMO patients is less common internationally despite data suggesting a high diagnostic yield [10–14].

The aim of this retrospective analysis of admission screening CT findings after cannulation for V-V ECMO was to (1) report the prevalence of extrapulmonary findings and (2) evaluate their impact on clinical management and ICU mortality. We hypothesized that extrapulmonary findings on screening CT would affect medical management and be associated with ECMO survival.

## Methods

### Participants

We included patients admitted to the adult ECMO ICU at St Thomas’ Hospital, London, UK between January 2011 and September 2021, who: (1) received V-V ECMO for severe refractory but potentially reversible respiratory failure; and (2) had a post-cannulation routine screening CT of the head (non-contrast), abdomen and pelvis (with contrast) performed at our centre. We excluded patients who received Veno-Arterial or hybrid ECMO modes.

### Study design

**Fig. 1:**
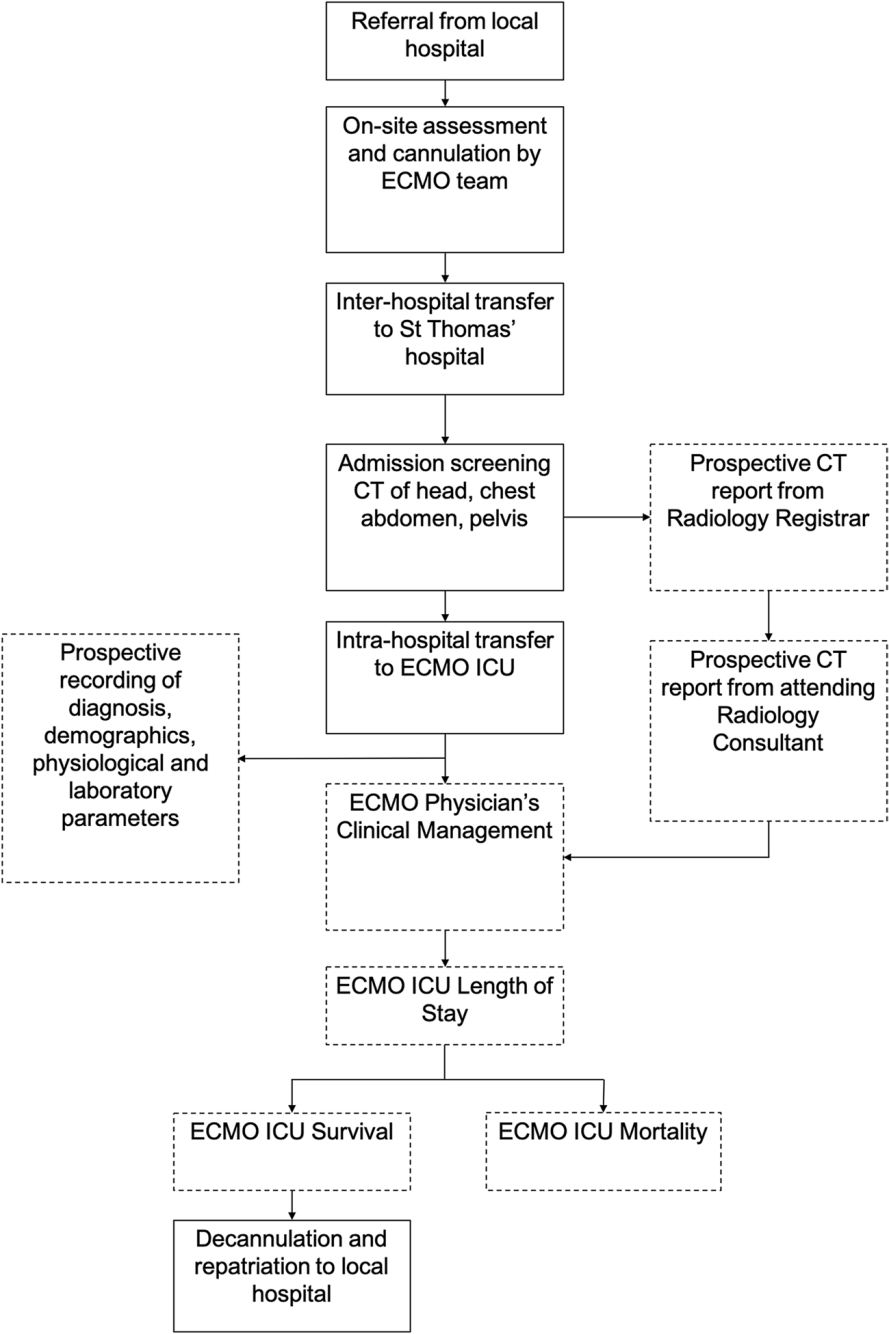
Processes of care at our centre. Dashed boxes indicate retrospectively evaluated data for inclusion in this study. CT denotes Computed Tomography. ECMO ICU denotes Extracorporeal membrane oxygenation. In the UK healthcare system, a registrar is a specialist trainee with a minimum of 4 years of postgraduate experience

This was a retrospective, observational study. Figure 1 outlines the standard processes of care at our centre [6, 7], and the retrospective screening methods. Briefly, routine screening CT scans were contemporaneously reported by (1) a radiology registrar (with a minimum of 4 year postgraduate experience) and (2) a consultant radiologist. A team of investigators (PC, LG, SK, VC, FP, DE) (blinded to outcome) reviewed all admission routine screening CT reports in the Picture and Archive Communication System (PACS; Advanced Data Systems Corporation, Paramus, New Jersey, USA) and identified incidental extrapulmonary findings according to the radiology report at the time of CT acquisition. CT imaging was not reinterpreted for this study and the same radiology reports and imaging were available to the treating physicians when patient care took place. In patients where there was known extrapulmonary pathology (e.g., pancreatitis as the cause of ARDS) this pathology was not considered an incidental finding. All data extraction on incidental findings was adjudicated by PC and LG, and in case of discordance a third member of the study team was identified to adjudicate. Next, we reviewed the electronic health record (ICIP; Philips, Eindhoven, the Netherlands and Electronic Patient Record) for evidence of (1) any documented invasive intervention prompted by an extrapulmonary CT finding; and (2) changes to the medical plan due to an extrapulmonary finding. We also extracted data on baseline demographics including the indication for V-V ECMO, admission physiological and laboratory variables and death occurring at our ECMO ICU. We defined mortality at this timepoint, since following decannulation from ECMO patients is routinely repatriated to the referring institution and, therefore, lost to follow up.

This study was approved as a service evaluation, as defined by the UK Health Research Authority, and received institutional approval (Authorization N14763) waiving the need for informed consent.

### Statistical analyses

We compared categorical variables using Chi Square tests; continuous variables were visually examined for normality, with normally distributed variables compared using t tests and non-normally distributed variables compared with Mann–Whitney *U* tests. Confidence intervals for proportions of incidental CT findings were calculated using the binomial exact calculation. We created multivariable logistic regression models to explore associations between CT incidental findings and ECMO ICU mortality. We created Kaplan–Meier curves to estimate and visualize survival functions and used log-rank test to compare the outcome of patients with incidental imaging findings, with discharge alive from the ECMO ICU considered a censoring event. All statistical analyses was carried out using R version 4.2.2 (R core team. R: a language and environment for statistical computing, Vienna, Austria). A two-sided *p* value < 0.05 was considered statistically significant. Variables with missing data were omitted from analyses.

We adhered to the STROBE (Strengthening the Reporting of Observational Studies in Epidemiology) statement guidelines [15].

## Results

### Patient population

**Fig. 2:**
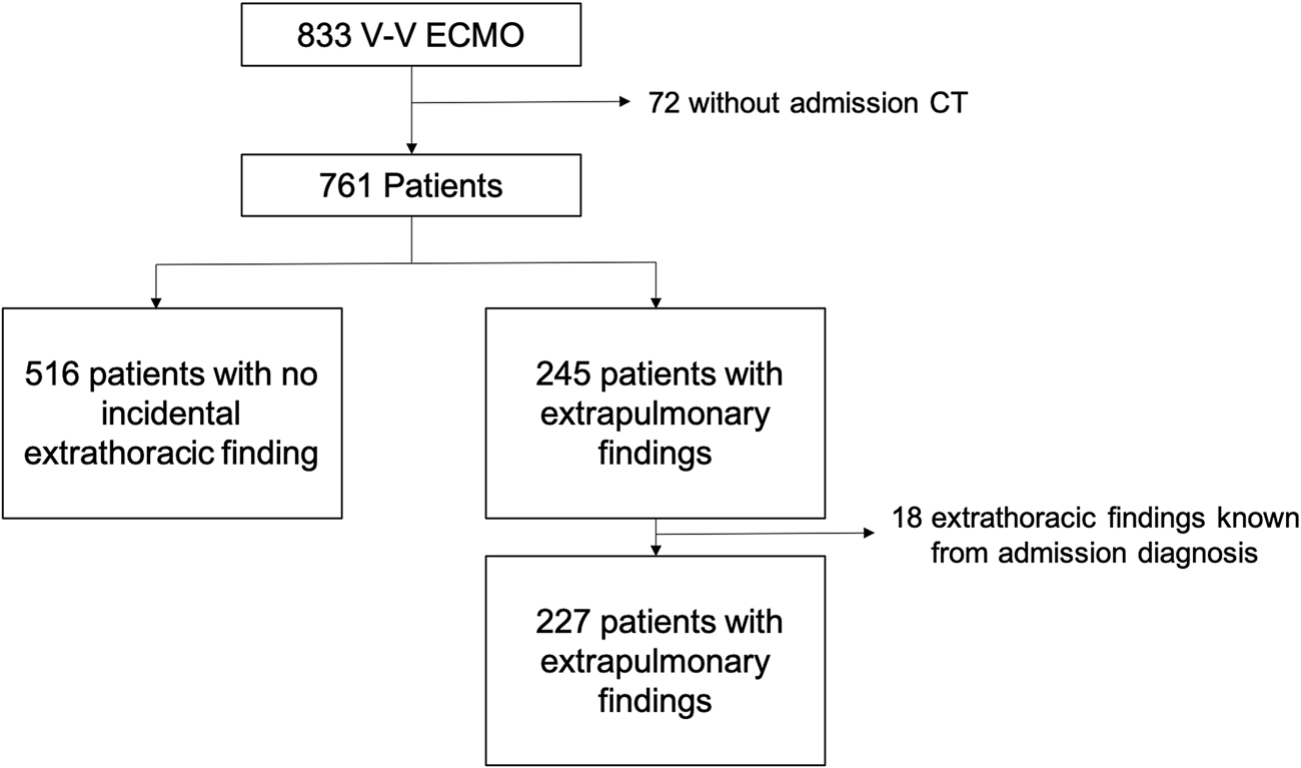
CONSORT diagram of the cohort. V-V ECMO denotes veno-venous extracorporeal membrane oxygenation. CT denotes computed tomography

During the study period (January 2011–June 2021), 833 patients with severe respiratory failure received V-V ECMO. Among these, 72 patients (8.6%) did not have an admission screening CT performed, thereby 761 patients (91.4%) were included in our analyses (see Fig. 2). Cohort characteristics are displayed in Table 1. Patients were commonly male (59%), median age 45 (34–53) years, with ARDS as the primary indication for V-V-ECMO. The ECMO ICU length of stay was 19 days (IQR 12–31 days); ECMO ICU mortality was 18.9%.

**Table 1:** Admission characteristics and subsequent outcomes

Variable	Total cohort (*N* = 761)
*Demographics*	
Age, years	45 (34–53)
Male sex, n(%)	449 (59)
Body mass index (kg/m^2^)	27.78 (24.2–33.6)
*Clinical parameters*	
APACHE II score	17 (14–21)
Peak pressure (cmH_2_O)	22 (20–27)
Positive end expiratory pressure (cmH_2_O)	10 (10–10)
Dynamic compliance (ml/cmH_2_O)	12.5 (5.6–21)
Heart rate (BPM)	97 (82–112.5)
Mean arterial pressure (mmHg)	80 (70–90)
Lactate (mmol/L)	1.95 (1.3–3.7)
Haemoglobin (g/L)	98 (87–112)
Platelets (× 10^9^/L)	180.5 (106.5–256)
International normalized ratio	1.1 (1–1.2)
Bilirubin (µmol/l)	13 (8–23)
Creatinine (µmol/l)	110.5 (64–192.75)
C-reactive protein (mg/L)	200 (87–315)
*Diagnostic category*	
Asthma, n(%)	41 (5.4)
Extrapulmonary ARDS, n(%)	54 (7.1)
Non-infectious pulmonary ARDS, n(%)	50 (6.6)
Pulmonary vasculitis	22 (2.9)
Inflammatory or interstitial lung disease	28 (3.7)
Infectious pulmonary ARDS, n(%)	532 (69.9)
Pneumonia (aspiration)	37 (4.9)
Pneumonia (bacterial)	222 (29.2)
Pneumonia (viral)	273 (35.9)
Other, n(%)	84 (11.0)
Trauma	25 (3.3)
Inhalation injury	8 (10.5)
Obstetrics	3 (0.4)
Pulmonary Embolism	7 (0.9)
Other diagnoses	41 (5.4)
*Outcome*	
ECMO ICU Length of stay (days)	19 (12–31)
ECMO ICU Mortality, n(%)	144 (18.9)

All data presented as median (interquartile range) or n(%) if specified

*APACHE* Acute Physiology and Chronic Healthy Evaluation, *ECMO ICU* Extracorporeal Membrane Oxygenation Intensive Care Unit

### Radiological findings

Incidental extrapulmonary findings are shown in Table 2. Of the 761 patients, 227 (29.8%) had at least one incidental extrapulmonary finding comprising intracranial (14.3%), intra-abdominal (17.6%) or both (2.1%). Intracranial haemorrhage and colitis were most common.

**Table 2:** Incidental extrapulmonary findings

Extrapulmonary findings (*N* = 761)	*n*(%)	95% CIs
*Total patients with extrapulmonary findings*	245 (32.2)	28.9,35.6
Known intra-abdominal pathology	18 (2.4)	N/A
Known intra-cranial pathology	3 (0.4)	N/A
Incidental extrapulmonary findings	227* (29.8)	26.6, 33.2
*Any incidental intracranial findings*	109 (14.3)	11.9, 17.0
Intracranial haemorrhage	67 (8.8)	6.9, 11.1
Cerebral oedema	42 (5.5)	4.0, 7.4
Cerebral infarction	18 (2.4)	1.4, 3.7
*Any incidental intra-abdominal finding*	134 (17.6)	15.0, 20.5
Sub-diaphragmatic bleeding	17 (2.2)	1.3, 3.6
Intra-abdominal bleeding	11 (1.4)	0.7, 2.6
Retroperitoneal hematoma	3 (0.4)	< 0.1, 1.15
Psoas hematoma	3 (0.4)	< 0.1, 1.15
Intra-abdominal collection	11 (1.4)	0.7, 2.6
Colitis	43 (5.7)	4.1, 7.5
Ischaemic colitis	8 (1.1)	0.5, 2.1
Splenic infarcts	21 (2.8)	1.7, 4.2
Liver infarcts	10 (1.3)	0.6, 2.4
Pancreatitis	9^†^ (1.2)	0.5, 2.2
Cholecystitis	23^‡^ (3.0)	1.9, 4.5
Organomegaly	1 (0.1)	< 0.1, 0.7
Ascites	38 (5.0)	3.6, 6.8
Subdiaphragmatic venous thrombosis	17 (2.2)	1.3, 3.6
ECMO cannula malposition	3 (0.4)	0.08, 1.15
Endotracheal tube malposition	21 (2.8)	1.7, 4.2

*3 patients with known intra-abdominal pathology had incidental intracranial findings. No patient with known intracranial pathology had an incidental intraabdominal finding. ^†^3 cases of radiological pancreatitis were deemed met the Atlanta criteria. ^‡^16 cases of cholecystitis met the Tokyo criteria, all acalculous

ECMO denotes extracorporeal membrane oxygenation

### Changes in clinical management following CT

A summary of clinical decision making prompted by incidental extrapulmonary findings is shown in Additional file 1: Table S1. Twelve of 227 patients with at least one incidental extrapulmonary finding (1.6%) underwent invasive interventions including exploratory laparotomy (six patients), imaging-guided percutaneous drainage (two patients) and endoscopic retrograde cholangiopancreatography, cholecystostomy, external ventricular drain placement and ECMO cannula manipulation (one patient each). Non-invasive changes in management, most commonly to anticoagulation, occurred in 119 patients (52.4%) (Additional file 1: Table S1).

### Association with outcome

**Fig. 3:**
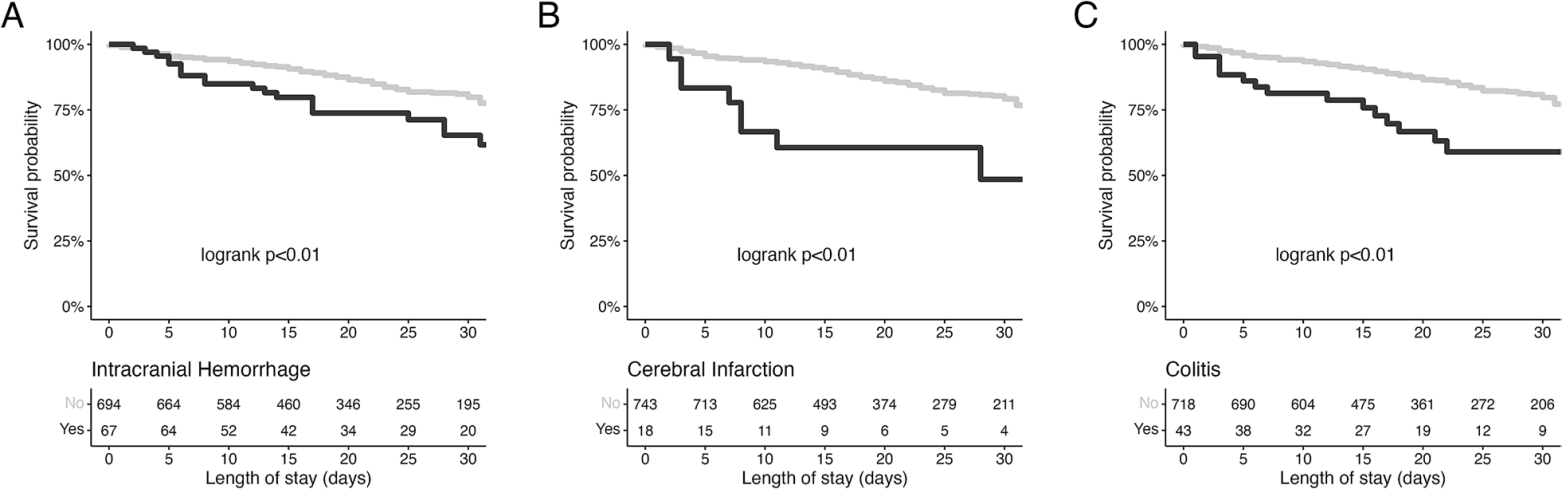
Kaplan–Meier curves of the association of ECMO ICU mortality with extrapulmonary imaging findings. **A** Survival curves for patients with and without Intracranial Haemorrhage. **B** Survival curves for patients with and without Cerebral Infarction. **C** Survival curves for patients with and without Colitis. *P* values are derived from the log-rank test. Statistical testing relates to ECMO ICU mortality but for clarity of visualization the curves are censored at day 30. ECMO ICU denotes extracorporeal membrane oxygenation intensive care unit

Three extrapulmonary incidental findings—intracranial haemorrhage, cerebral infarction and colitis—demonstrated an association with ECMO centre mortality using univariable logistic regression (see Table 3). These three incidental findings remained associated with mortality when adjusted for age, sex, and APACHE II score. Figure 3 displays the Kaplan–Meier curves showing higher risk of death for patients with intracranial haemorrhage, cerebral infarction or colitis. A sensitivity analysis excluding patients with probable ischaemic colitis (8/43 patients with colitis (18.6%) found a similar magnitude of association with mortality (see Additional file 1: Table S2).

**Table 3:** Univariable and adjusted (for age, sex and APACHE II score) regression modelling for extrapulmonary imaging findings and ECMO ICU mortality

Variable	Univariable OR (95% CI)	*P* value	Adjusted OR (95% CI)	*P* value
Age	1.05 (1.03, 1.07)	< 0.01	–	–
Sex (M)	1.82 (1.24, 2.70)	< 0.01	–	–
APACHE II score	1.10 (1.05, 1.14)	< 0.01	–	–
Intracranial haemorrhage	2.29 (1.31, 3.92)	< 0.01	2.34 (1.31, 4.12)	< 0.01
Cerebral infarction	3.57 (1.34, 9.22)	< 0.01	3.59 (1.26, 9.86)	0.01
Colitis	2.45 (1.24, 4.65)	< 0.01	2.80 (1.35, 5.67)	< 0.01

*OR* (*95% CI*) odds ratio (95% confidence interval)

*APACHE* Acute Physiology and Chronic Healthy Evaluation

### Association with admission physiological and laboratory values

Table 4 displays physiologic and laboratory parameters of patients with and without colitis. Patients with colitis identified in screening CT had a higher APACHE II score, lower dynamic compliance, more hypotension and tachycardia, and higher bilirubin, international normalized ratio, creatinine and lactate levels, indicating a worse shock state. These associations were not seen with other incidental imaging findings (Additional file 1: Tables S3–S4).

**Table 4:** Characteristics of patients with colitis

Variable	Without colitis (*n* = 718)	With colitis (*n* = 43)	*P* value
Age, years	45 (34–53)	41 (29–52)	0.30
Male sex, *n*(%)	428 (60)	21 (49)	0.20
Body mass index (kg/m^2^)	28.0 (24.0–34.0)	24.0 (23.0–29.0)	< 0.01
APACHE II Score	17 (14–20)	20 (18–23)	< 0.01
Peak pressure (cmH_2_O)	22 (20–27)	21 (20–25)	0.34
Positive end expiratory pressure (cmH_2_O)	10 (10–12)	10 (8.5–10)	0.11
Dynamic compliance (ml/cmH_2_O)	13.0 (6.1–21.2)	10.0 (1.6–17.5)	0.03
Heart rate (BPM)	96 (81–111)	113 (95.5–118)	< 0.01
Mean arterial pressure (mmHg)	80 (70–90)	73 (64–81)	< 0.01
Lactate (mmol/L)	1.9 (1.3–3.5)	6.6 (1.6–10.1)	< 0.01
Haemoglobin (g/L)	98 (87–112)	99 (85–116.5)	0.90
Platelets (× 10^9^/L)	183 (113–258)	83 (39–145)	< 0.01
International normalized ratio	1.1 (1.0–1.2)	1.5 (1.1–2.0)	< 0.01
Bilirubin (µmol/l)	13 (8–22)	20 (10–36.5)	0.01
Creatinine (µmol/l)	107 (64–190)	162 (94.5–231)	0.02
C-reactive protein (mg/L)	207 (64–190)	185 (80–265)	0.23
ECMO ICU length of stay, days	20 (12–31)	19 (9.5–25)	0.14
ECMO ICU mortality, *n*(%)	129 (18.0)	15 (34.8)	< 0.01

Data presented as median (interquartile range) unless specified

APACHE denotes Acute Physiology and Chronic Healthy Evaluation. ECMO ICU denotes Extracorporeal Membrane Oxygenation Intensive Care Unit

## Discussion

In this large retrospective cohort of patients receiving V-V ECMO who had a routine screening CT on admission following cannulation, around one-third had at least one incidental extrapulmonary finding. These findings often led to a change in medical management, although few patients underwent an invasive procedure (Additional file 1: Table S1). Incidental extrapulmonary findings of colitis, intracranial haemorrhage and cerebral infarction were associated with greater risk of mortality.

### Diagnostic yield

Important considerations in the interpretation of our study are the large sample size, accumulated over 10 years, and the consistent use of a routine screening CT scan. In a recent international survey, only 12.5% of centres routinely performed neuroimaging following cannulation for V-V ECMO [16]. It is likely that a routine total body CT is even less common. Two previous single-centre studies have reported the findings from routine use of whole-body CT but had substantially smaller sample sizes (*N* = 65 [10], *N* = 198 [11]) with one cohort that did not routinely utilize intravenous contrast [11]. Several other single centre studies reported prevalence and clinical impact of CT imaging during treatment with V-V ECMO, but do not use a routine screening CT, have smaller sample sizes, and contain mixed adult and pediatric populations [17–20]. Larger studies from the Extracorporeal Life Support Organization Registry focus specifically on the risk factors and prevalence of neurologic complications during V-V ECMO [21–24], but again do not reflect the routine use of screening CT.

The concept of ‘testing threshold’ has been proposed as an approach to determining the appropriateness of a clinical investigation [25], including systematic screening. The following equation uses the benefits of testing (when linked to a resulting change in treatment) as the denominator with the risks of testing as the numerator:

$$ {\text{Testing Threshold}} = \frac{{\left( {1 - {\text{Specificty}}} \right) \times Risk_{Treatment} + Risk_{Test} }}{{\left( {1 - {\text{Specificity}}} \right) \times  Risk_{Treatment} + Sensitivity \times Benefits_{Treatment} }} $$

When the point prevalence of the condition exceeds this ‘testing threshold’ then the use of a screening test is justifiable [25]. The prevalence of potentially serious incidental CT findings in our cohort compares favorably to the testing or risk thresholds proposed to justify CT examinations of adults presenting to an emergency department with possible ischaemic bowel (2.1%) [26], pulmonary embolism (1.4–1.8%) [27, 28], aortic dissection (0.6%) [29] or minor traumatic brain injury (0.3–0.4%) [30] or used to justify CT in outpatients for coronary artery disease (7–15%) [31, 32] or as screening for lung cancer (0.4–3.4%) [33]. However, a key consideration is the risks of screening CT including the need for transport of critically ill patients. Although the rate of life-threatening adverse events during transfer to CT is reported as high as 7% in one prospective study [34], safe transfer is reported in multiple other studies [7, 10, 11, 16, 17, 35]. At our centre there has been no serious incident of avoidable harm reported from the transfer of a V-V ECMO patient for diagnostic imaging. However, the safety of transfer for ECMO patients for CT would be best assessed in a dedicated prospective study. Centres newly adopting this practice should consider standardising transfer processes, adopt dedicated training for staff and consider ongoing safety audit.

### Intracranial findings

We found a higher prevalence of both intracranial haemorrhage (prevalence of 8.8% versus 2.8%) and cerebral infarction (prevalence of 2.4% versus 1.2%) on routine screening CT compared to the most recent ELSO database study (*n* = 7579) [22]. Furthermore, we found a lower mortality rate than reported from the ELSO database (intracranial haemorrhage 32.8% versus 73.2%); cerebral infarction 44.4% versus 73.9%) [22]. This difference may relate to the use of routine screening CT which can identify pathology prior to the development of overt clinical signs and potentially lead to changes in management which may influence pathology progression.

### Intraabdominal findings

Two studies reported incidental intraabdominal pathology following a screening CT in V-V ECMO patients with a prevalence of 16.9% [10] to 27% [11] similar to our findings (17.6%). A novel result of our study is the association of colitis with mortality when adjusted for age, sex and APACHE II score with similar findings in a sensitivity analysis removing patients with probable ischaemic colitis. Patients with colitis had worse shock and multiple organ failure. This may suggest that radiological colitis is a marker of abnormal tissue perfusion and congestion resulting from heart–lung interactions in severe ARDS (‘shock bowel’ [36]).

### Clinical consequences

As well as affecting the ultimate outcome, incidental extrapulmonary findings impacted the early clinical management of our patients, with the majority having some changes in their medical treatment, and only few undergoing invasive interventions. However, the impact of screening CT on decision making may be influenced by patient-specific characteristics and local practices. For example, changes to anticoagulation were the most common medical decision prompted by extrapulmonary findings, but decisions might be influenced by the presence of pulmonary embolism, which had a prevalence of 15.8% in this cohort at admission CT (see Additional file 1: Table S5 for the prevalence of incidental intrathoracic findings). In addition, although 14.3% of the cohort had intracranial pathology, only one patient underwent a neurosurgical intervention; this might reflect earlier recognition but may be influenced by the need for interhospital transfer given the absence of neurosurgical services at our centre.

### Limitations and strengths

Our study has a number of limitations beyond its single centre retrospective nature. First, retrospective adjudication of medical decision making relating to CT findings is challenging as it relies upon interpretation of documentation which may be incomplete. Moreover, although our sample size is relatively large for an ECMO cohort, it may be underpowered with respect to associations of imaging findings of low prevalence with mortality. The absence of a control group who did not receive universal screening CT is a limitation of this study. However, our aims were to establish the prevalence of extrapulmonary findings and their relationship with care and outcomes rather than the superiority of systematic imaging (which would require prospective randomisation). Finally, there may be unmeasured confounders which also influence decision-making or outcome. Our data set, study design and power does not allow for a meaningful answer on the reduction of risk potentially conferred by a management strategy which includes a screening CT. Whether performing routine CT affects outcome and by how much remains to be determined.

Strengths of our study are the comparatively large sample size reflecting over 10 years of patients managed at a high volume ECMO centre covering a population base of 18 million people. The number of mortality events (144) allowed the use of multivariable regression modelling enabling associations between screening CT incidental findings and outcome. Furthermore, the routine use of screening CT means that our results may give a reasonable estimate of the true prevalence of extrapulmonary findings in this population.

## Conclusions

A routine screening CT of the head, abdomen and pelvis following cannulation for V-V ECMO frequently identifies incidental extrapulmonary findings which often change clinical management and are prognostic of outcome. We suggest ECMO centres consider a routine screening CT for all patients following cannulation for V-V ECMO. The utility of this approach may be influenced by a centre’s case volume, selection criteria for V-V ECMO and experience of transporting patients receiving extracorporeal support.

### Supplementary Information

**Additional file 1:** Additional Tables, Tables S1-S5.

## Data Availability

Deidentified data can be made available from the corresponding author upon reasonable request. A data use agreement will be required before the release of data with institutional review board approval as appropriate.

## Abbreviations

APACHE: Acute Physiology and Chronic Healthy Evaluation
ARDS: Acute respiratory distress syndrome
CT: Computed tomography
ICU: Intensive care unit
IQR: Interquartile range
OR: Odds ratio
STROBE: Strengthening the reporting of observational studies in epidemiology
V-V ECMO: Veno-venous extracorporeal membrane oxygenation
