# Supplementary material for: Clinical impact of screening computed tomography in extracorporeal membrane oxygenation: a retrospective cohort study
Source: Ann Intensive Care. 2023 Sep 26;13:90. doi: 10.1186/s13613-023-01187-w (PMC10522559; doi:10.1186/s13613-023-01187-w)
Supplement: Supplementary file 1 — Additional file 1: Additional Tables, Tables S1-S5. [file 13613_2023_1187_MOESM1_ESM.docx]

**Clinical Impact of Screening Computed Tomography in Extracorporeal Membrane Oxygenation**

Patrick D Collins MD^1^*, Lorenzo Giosa MD^1,2^*, Sushil Kathar MD^1^, Valentina Camarda MD^1^, Filippo Palmesino MD^1^, Darshan Eshwar MD^1^, Nicholas A Barrett MD^1,2^, Andrew Retter MD^1^, Francesco Vasques MD^1^, Barnaby Sanderson BSc^1^, Sze M Mak MD^3^, Louise Rose PhD^4^, Luigi Camporota, MD, PhD^1,2^.

**Online Data Supplement**

| **Supplemental Table E1: Influence on clinical management** | |
| --- | --- |
| **Medical Interventions** | **Incidental Extrapulmonary Findings (N=227)** |
| **Invasive procedure** | 12 (5.3) |
| External ventricular drain | 1 (0.4) |
| Laparotomy +/- | 6 (2.6) |
| Endoscopic retrograde cholangiopancreatography | 1 (0.4) |
| Cholecystostomy | 1 (0.4) |
| ECMO cannula repositioning | 1 (0.4) |
| Imaging guided percutaneous drainage | 2 (0.9) |
| **Change in medical management** | 119 (52.4) |
| Specialist consult or further imaging | 10 (4.4) |
| Anticoagulation change of target/cessation | 96 (42.3) |
| Change to transfusion threshold target (eg platelets) | 6 (2.6) |
| Change to nutritional strategy | 22 (9.7) |
| Osmotherapy | 2 (0.9) |
| Neuroprotection | 37 (16.3) |
| Antiplatelet institution | 1 (0.4) |
| Prophylactic anti-epileptic drug | 3 (1.3) |
| Withdrawal of care | 1 (0.4) |
| Parenteral steroids* | 1 (0.4) |
| All data presented as, n(%).  *One patients admission CT raised concerns for the syndrome of Waterhouse Friderichsen | |

| **Supplemental Table E2: Extrapulmonary findings as predictors of ECMO ICU Mortality on univariable logistic regression** | | |
| --- | --- | --- |
| **Imaging Variable** | **OR (95% CI)** | **P Value** |
| Intracranial Haemorrhage | 2.29 (1.31, 3.92) | <0.01 |
| Cerebral oedema | 1.07 (0.48, 2.19) | 0.90 |
| Cerebral Infarction | 3.57 (1.34, 9.22) | <0.01 |
| Intra-abdominal collection | 0.85 (0.13, 3.29) | 0.80 |
| Colitis | 2.45 (1.24, 4.65) | <0.01 |
| Colitis (excluding ischaemic colitis) | 2.35 (1.10, 4.75) | 0.02 |
| Ischaemic colitis | 2.60 (0.53, 10.7) | 0.20 |
| Pancreatitis | 0.71 (0.16, 2.13) | 0.60 |
| Cholecystitis | 0.38 (0.06, 1.31) | 0.20 |
| Acute subdiaphragmatic bleeding | 0.00 (0.00, ) | >0.99 |
| Abdominal venous thrombosis | 0.85 (0.20, 2.63) | 0.80 |
| Organomegaly | 0.00 (0.00, ) | >0.99 |
| Ascites | 0.72 (0.27, 1.63) | 0.50 |
| Splenic infarcts | 1.74 (0.61, 4.38) | 0.30 |
| Liver infarcts | 1.07 (0.16, 4.33) | >0.90 |

OR (95% CI) denotes Odds Ratio (95% Confidence Interval).

| **Supplemental Table E3: Characteristics of patients with intracranial haemorrhage** | | | |
| --- | --- | --- | --- |
| **Variable** | **No Intracranial Haemorrhage (n=694)** | **Intracranial Haemorrhage**  **(n=67)** | **P value for difference** |
| Age, years | 45 (34-53) | 47 (37-53.5) | 0.28 |
| Male sex, n(%) | 409 (58.9) | 40 (59.7) | >0.99 |
| Body mass index (kg/m^2^) | 27.9 (24.5-34.0) | 26.3 (22.7-30.9) | <0.01 |
| APACHE II Score | 17 (14-21) | 18 (14-20) | 0.85 |
| Peak pressure (cmH_2_O) | 22 (20-27) | 20 (20-26) | 0.13 |
| Positive end expiratory pressure (cmH_2_O) | 10 (10-11) | 10 (10-10) | 0.31 |
| Dynamic compliance (ml/cmH_2_O) | 12.5 (5.7-21.1) | 13.5 (4.8-18.9) | 0.68 |
| Heart rate (BPM) | 97 (82-113) | 93 (79.5-107.5) | 0.32 |
| Mean arterial pressure (mmHg) | 79 (69.75-89) | 82 (73.5-98) | 0.01 |
| Lactate (mmol/L) | 1.9 (1.3-3.675) | 2 (1.225-3.675) | 0.99 |
| Haemoglobin (g/L) | 98 (88-113) | 93.5 (83.25-102) | <0.01 |
| Platelets (x10^9^/L) | 183 (110-260.5) | 157 (80-235) | 0.02 |
| International normalized ratio | 1.1 (1-1.2) | 1.1 (1-1.2) | 0.91 |
| Bilirubin (µmol/l) | 13 (8-23) | 15 (10-24) | 0.10 |
| Creatinine (µmol/l) | 112 (64-191.5) | 107 (74.5-209.5) | 0.43 |
| C-reactive protein (mg/L) | 198 (84-312.5) | 231 (150.5-330.5) | 0.06 |
| ECMO ICU length of stay, days | 19 (12-31) | 20 (12-31) | 0.89 |
| ECMO ICU mortality, n(%) | 122 (17.6) | 22 (32.8) | <0.01 |
| Data presented as median (interquartile range) unless specified.  APACHE denotes Acute Physiology and Chronic Healthy Evaluation. ECMO ICU denotes Extracorporeal Membrane Oxygenation Intensive Care Unit. | | | |

| **Supplemental Table E4: Characteristics of patients with cerebral infarction** | | | |
| --- | --- | --- | --- |
| **Variable** | **No cerebral infarction (n=743)** | **Cerebral infarction**  **(n=18)** | **P value for difference** |
| Age, years | 45 (34-53) | 44 (35.5-54.75) | 0.50 |
| Male sex, n(%) | 437 (58.8) | 12 (66.7) | 0.67 |
| Body mass index (kg/m^2^) | 27.8 (24.2-33.6) | 27.2 (24.3-32.7) | 0.62 |
| APACHE II Score | 17 (14-21) | 16 (13.25-20) | 0.47 |
| Peak pressure (cmH_2_O) | 22 (20-27) | 20 (20-25.5) | 0.29 |
| Positive end expiratory pressure (cmH_2_O) | 10 (10-12) | 10 (10-10) | 0.58 |
| Dynamic compliance (ml/cmH_2_O) | 12.6 (5.6-21.1) | 11.6 (5.5-20) | 0.96 |
| Heart rate (BPM) | 97 (82-112) | 101 (87.5-118.25) | 0.24 |
| Mean arterial pressure (mmHg) | 79 (70-90) | 80 (70-87) | 0.95 |
| Lactate (mmol/L) | 2 (1.3-3.7) | 1.9 (1.4-3.2) | 0.97 |
| Haemoglobin (g/L) | 98 (87-112) | 107 (93-116.8) | 0.34 |
| Platelets (x10^9^/L) | 181 (103.5-256) | 169 (136-255) | 0.81 |
| International normalized ratio | 1.1 (1-1.2) | 1.25 (1.1-1.3) | 0.02 |
| Bilirubin (µmol/l) | 13 (8-23) | 12.5 (8.5-20.75) | >0.99 |
| Creatinine (µmol/l) | 110 (64-192.25) | 123 (77.25-203.75) | 0.64 |
| C-reactive protein (mg/L) | 204 (87-315) | 163.5 (86.5-287.25) | 0.76 |
| ECMO ICU length of stay, days | 20 (12-31) | 13 (8-26.75) | 0.16 |
| ECMO ICU mortality, n(%) | 136 (18.3) | 8 (44.4) | 0.01 |
| Data presented as median (interquartile range) unless specified.  APACHE denotes Acute Physiology and Chronic Healthy Evaluation. ECMO ICU denotes Extracorporeal Membrane Oxygenation Intensive Care Unit. | | | |

| **Supplemental Table E5: Incidental Findings on thoracic CT** | | | | |
| --- | --- | --- | --- | --- |
| **Incidental Thoracic Findings** | | | | **Total Cohort (N=761)** |
| Only parenchymal abnormalities* | | | | 226 (29.7) |
| Extra-parenchymal findings | | |  | 409 (53.7) |
|  | Pulmonary embolism | | | 120 (15.8) |
|  |  | Main pulmonary artery | | 30 (3.9) |
|  |  | Segmental | | 85 (11.2) |
|  |  | Sub-segmental | | 36 (4.7) |
|  | Pleural effusion | | | 211 (27.7) |
|  | Haemothorax | |  | 4 (0.5) |
|  | Pneumothorax | | | 88 (11.6) |
|  | Isolated pneumomediastinum | | | 53 (7.0) |
|  | Pericardial effusion | | | 17 (2.2) |
|  | Right ventricular strain | | | 44 (5.8) |
|  | Rib or sternal fractures | | | 14 (1.8) |
| All data presented as n(%).  *Parenchymal changes due to the underlying disease were not evaluated in this analysis. | | | | |
